# Conversion of time-varying Stokes coefficients into mass anomalies at the Earth’s surface considering the Earth’s oblateness

**DOI:** 10.1007/s00190-018-1128-0

**Published:** 2018-02-19

**Authors:** Pavel Ditmar

**Affiliations:** Delft University of Technology, Stevinweg 1, 2628 CN Delft, The Netherlands

**Keywords:** Stokes coefficients, Spherical harmonics, Time-varying gravity, Mass transport, GRACE, GRACE Follow-On

## Abstract

Time-varying Stokes coefficients estimated from GRACE satellite data are routinely converted into mass anomalies at the Earth’s surface with the expression proposed for that purpose by Wahr et al. (J Geophys Res 103(B12):30,205–30,229, 1998). However, the results obtained with it represent mass transport at the spherical surface of 6378 km radius. We show that the accuracy of such conversion may be insufficient, especially if the target area is located in a polar region and the signal-to-noise ratio is high. For instance, the peak values of mean linear trends in 2003–2015 estimated over Greenland and Amundsen Sea embayment of West Antarctica may be underestimated in this way by about 15%. As a solution, we propose an updated expression for the conversion of Stokes coefficients into mass anomalies. This expression is based on the assumptions that: (i) mass transport takes place at the reference ellipsoid and (ii) at each point of interest, the ellipsoidal surface is approximated by the sphere with a radius equal to the current radial distance from the Earth’s center (“locally spherical approximation”). The updated expression is nearly as simple as the traditionally used one but reduces the inaccuracies of the conversion procedure by an order of magnitude. In addition, we remind the reader that the conversion expressions are defined in spherical (geocentric) coordinates. We demonstrate that the difference between mass anomalies computed in spherical and ellipsoidal (geodetic) coordinates may not be negligible, so that a conversion of geodetic colatitudes into geocentric ones should not be omitted.

## Introduction

Since the launch of Gravity Recovery and Climate Experiment (GRACE) satellite mission in 2002 (http://www.csr.utexas.edu/grace), satellite gravimetry has become one of the key tools to study large-scale mass transport in the Earth’s system. Mass transport estimates based on GRACE data have been successfully applied in numerous studies of the solid Earth, cryosphere, oceans, and continental water resources (for an overview, see, e.g., Wouters et al. 2014). To produce these estimates, one exploits information about temporal gravity variations sensed by GRACE satellites.

Newton’s law of gravitational attraction allows temporal gravity field variations to be computed uniquely, as soon as mass anomalies are given (see, e.g., Chao et al. 1987). Such a relationship is used, for instance, to estimate rapid gravity field variations caused by non-tidal mass transport in the ocean and atmosphere. These estimates are distributed as a so-called Atmosphere and Ocean De-aliasing product AOD1B (Dobslaw et al. 2016), which is needed, along with other background force models, for a preprocessing of GRACE data. This is because rapid mass transport signals cannot be properly interpreted and play a role of additional source of noise in GRACE data. To properly process mass re-distribution in the atmosphere, it is essential to take into account its vertical structure (Swenson and Wahr 2002; Flechtner 2007), as well as the oblateness of the Earth (Forootan et al. 2013). All these considerations are taken into account in the production of the latest release (RL06) of the AOD1B product (Dobslaw et al. 2016).

The inverse problem—conversion of temporal gravity field variations into mass anomalies—is more difficult. It cannot be solved uniquely without additional assumptions about the mass transport. A commonly used assumption is that mass transport takes place at a sphere of a certain radius *a*. Then, the Stokes coefficients describing the time-varying gravity field can be uniquely related to Fourier coefficients of mass anomalies, provided that the corresponding base functions are defined as surface spherical harmonics (Chao et al. 1987). Wahr et al. (1998) proposed to apply such an approach to recovery of mass anomalies from GRACE data, and this has been done since then in hundreds of studies.

Unfortunately, the overwhelming majority of authors ignore the fact that the actual Earth’s surface substantially deviates from the sphere. If the radius *a* is defined as the mean equatorial radius or the semimajor axis of the reference ellipsoid (which is typically the case), these deviations become particularly large in polar areas. Obviously, such deviations may result in some distortions in mass anomaly estimates (Chao 2005, 2016). However, they are not quantified so far in the context of GRACE-based estimates, the temporal resolution of which is limited to about 400 km (in terms of wavelengths).

Furthermore, the commonly used expressions for a conversion of Stokes coefficients into mass anomalies refer to *spherical* (geocentric) coordinates of a point, including the geocentric colatitude $\theta$. However, only a few authors define the exploited coordinates properly. In most of cases, $\theta$ is defined as just “the colatitude.” We consider this as an indication that the step of converting standard ellipsoidal (geodetic) geographical coordinates into spherical ones is likely omitted in many studies, especially if they are conducted by non-geodesists. Though the difference between geocentric and geodetic colatitudes is relatively minor $(\lesssim ~11.5^{\prime } \approx 21\,\text{ km })$, it might be improper to ignore it in all cases.

The primary goals of this publication are: (i) to demonstrate that mass anomaly estimates produced with the expressions from (Wahr et al. 1998) may not be sufficiently accurate (in particular, this concerns long-term mass losses in polar areas); (ii) to show that a simple modification of them may increase the accuracy by an order of magnitude; and (iii) to demonstrate that an (erroneous) interpretation of spherical coordinate $\theta$ as a geodetic colatitude may have a non-negligible effect onto the estimated mass anomalies.

It is important to stress that our discussion is limited to a recovery of a 2-D mass re-distribution. In practice, this means that our focus is on hydrology, ice sheets, and oceans. The corresponding mass variations take place at the Earth’s surface or just below it (at a depth not exceeding in most cases a few hundreds of meters). Then, our assumption that mass transport is confined to a thin layer near the Earth’s surface is fully justified, and a quantification of it in terms of equivalent water heights is physically meaningful.

Mass transport in the solid Earth definitely occurs deeper, spanning a much larger range of depths. For instance, hypocenters of large earthquakes are typically located at the depth of a few tens of km, whereas glacial isostatic adjustment (GIA) takes place in the asthenosphere, the top boundary of which is located in most places at the depth of 100–200 km. Therefore, the techniques discussed in this paper are not applicable to mass transport in the solid Earth. A more extended discussion of limitations associated with a recovery of 3-D mass transport processes from GRACE data can be found in Chao (2016).

The structure of this paper is as follows. In Sect. 2, we present a general expression for the computation of mass anomalies at the Earth’s surface, as well as possible simplifications of that expression, depending on the assumption about the Earth’s surface shape. That section concludes by a comparison of the proposed simplifications using real GRACE data, which allows us to identify the expression that is the most appropriate in practice. In Sect. 3, we address the issue of the proper definition of the coordinate $\theta$. Section 4 is left for a discussion and conclusions.

## Estimation of mass anomalies under different assumptions about the Earth surface geometry

### Theory

#### General information

Information delivered by GRACE is usually provided to the Earth science community in the form of monthly sets of Stokes coefficients. Those coefficients represent the mean value of the Earth’s gravitation potential $U (r,\theta ,\phi ,t)$ within a given month. The subtraction of a long-term mean value from each coefficient results in a time series of its temporal variations. Those variations can be linked to temporal variations $\Delta U (r,\theta ,\phi )$ of the gravitational potential (see, e.g., Heiskanen and Moritz 1967, Eqs. 2–39):

1 $$\begin{aligned} \Delta U (r,\theta ,\phi )= & {} \frac{GM_{\text {E}}}{a} \, \sum _{l=1}^{L_{\max }} \left( \frac{a}{r} \right) ^{l+1} \sum _{m=0}^{l} \left[ \Delta \bar{C}_{lm} \bar{P}_{lm}(\theta ) \cos m\phi \right. \nonumber \\&\left. +\, \Delta \bar{S}_{lm} \bar{P}_{lm}(\theta ) \sin m\phi \right] , \end{aligned}$$

where $(r,\theta ,\phi )$ are spherical coordinates of a given point (radial distance, colatitude, and longitude) in the terrestrial reference frame; *G* is the universal gravitational constant; $M_{\text {E}}$ is the Earth’s mass; *a* is the Earth’s radius (a more specific definition of this parameter is addressed below); *l* and *m* are the spherical harmonic degree and order, respectively; $L_{\max }$ is a model-specific maximum degree; $\Delta C_{lm}$ and $\Delta S_{lm}$ are temporal variations of Stokes coefficients (by definition, $\Delta S_{lm} = 0$ for $m = 0$); and $\bar{P}_{l,m}$ are normalized associated Legendre functions. The argument of time is omitted in Eq. (1) for the sake of brevity. The summation does not contain the degree-0 term, since variations in the total mass of the Earth (which are described by that term) are negligible.

The exact definition of the Earth’s radius *a* is a matter of convention. Classically, this parameter is defined as the semimajor axis of the reference ellipsoid or the mean Earth’s equatorial radius: $a \sim 6{,}378{,}136$ m (Heiskanen and Moritz 1967). GRACE data products are also provided in line with this definition. On the other hand, many publications define *a*, explicitly or implicitly, as the mean radius of the entire Earth: $a = 6371$ km (e.g., Chao et al. 1987; Wahr et al. 1998; Swenson and Wahr 2002). Strictly speaking, this means that an application of the expressions derived in those publications requires the corresponding rescaling of the GRACE-based Stokes coefficients. To the best of the author’s knowledge, however, this is never done in practice. Apparently, the impact of this rescaling is assumed to be minor. In any case, in all the derivations presented below, *a* is defined as the semimajor axis of the reference ellipsoid.

To make the further derivations simpler, we rewrite Eq. (1) in a more compact form:

2 $$\begin{aligned} \Delta U (r,\theta ,\phi ) = \frac{GM_{\text {E}}}{a} \, \sum _{l=1}^{L_{\max }} \left( \frac{a}{r} \right) ^{l+1} \sum _{m=-l}^{l} \Delta C_{lm} \bar{Y}_{lm}(\theta ,\phi ). \end{aligned}$$

In this notation, spherical harmonic order *m* runs from $-l$ to *l* and $\bar{Y}_{lm}$ are $4\pi$-normalized surface spherical harmonics:

3 $$\begin{aligned} \bar{Y}_{lm} (\theta ,\phi ) = \bar{P}_{l,|m|} (\cos \theta ) \left\{ \begin{array}{ll} \cos m\phi &{}\quad (m \ge 0) \\ \sin (-m \phi ) &{}\quad (m < 0). \\ \end{array} \right. \end{aligned}$$

The temporal variations $\Delta C_{lm}$ of Stokes coefficients in Eq. (2) are related to the traditionally considered ones as

4 $$\begin{aligned} \Delta C_{lm} = \left\{ \begin{array}{ll} \Delta \bar{C}_{lm} &{}\quad (m \ge 0) \\ \Delta \bar{S}_{lm} &{}\quad (m < 0). \\ \end{array} \right. \end{aligned}$$

In the further derivations, we make use of the publication by Swenson and Wahr (2002) as a starting point. We also use the same notation, when possible.

The general expression that connects temporal variations of density $\Delta \rho (r,\theta ,\phi )$ at/inside the Earth with temporal variations of Stokes coefficients is:

5 $$\begin{aligned} \Delta C_{lm} = \frac{a^2}{M_{\text {E}}} \, \frac{1}{(2l+1)} \int \int \limits _{ \Omega } \Delta I_l (\theta ,\phi ) \, \bar{Y}_{lm} (\theta ,\phi ) \, \hbox {d} \Omega , \end{aligned}$$

where integration covers the entire sphere:

6 $$\begin{aligned} \int \int \limits _{ \Omega } \hbox {d} \Omega = \int _0^{2\pi } \hbox {d}\phi \, \int _0^{\pi } \sin \theta \, \hbox {d}\theta , \end{aligned}$$

whereas $\Delta I_l (\theta ,\phi )$ describes vertically integrated density variations:

7 $$\begin{aligned} \Delta I_l (\theta ,\phi ) = \int _{0}^{\mathrm{top\, of\, atmos}} \left( \frac{r}{a} \right) ^{l+2} \Delta \rho (r,\theta ,\phi ) \, \hbox {d}r. \end{aligned}$$

Equation (5) is virtually equivalent to Eq. (2) in Swenson and Wahr (2002), with the exception that we watch the difference between the mean Earth’s radius and the equatorial one.

As it is explained in Sect. 1, we assume that mass transport takes place in a thin layer at the Earth surface. As such, it can be represented by a single mass layer, so that $\Delta I_l (\theta ,\phi )$ can be approximated as

8 $$\begin{aligned} \Delta I_l (\theta ,\phi )= & {} \int _{\mathrm{thin\, layer}} \left( \frac{r_s(\theta ,\phi )}{a} \right) ^{l+2} \Delta \rho (r,\theta ,\phi ) \, \hbox {d}r \nonumber \\&\approx \left( 1 + \frac{\xi + h}{a} \right) ^{l+2} \Delta \sigma (\theta ,\phi ) \end{aligned}$$

[cf. Eq. (7) in Swenson and Wahr (2002)]. In Eq. (8), $\Delta \sigma (\theta ,\phi )$ are variations of surface density (i.e., mass variations per unit area) and $r_s(\theta ,\phi )$ is the function describing the shape of the Earth’s surface that can be represented with a high accuracy as

9 $$\begin{aligned} r_s(\theta ,\phi ) = a + \xi (\theta ,\phi ) + h(\theta ,\phi ) \end{aligned}$$

with $\xi (\theta ,\phi )$ being the height of the geoid above the sphere of radius *a*, whereas $h(\theta ,\phi )$ is the orthometric height of the Earth’s surface topography [cf. Eq. (5) in Swenson and Wahr (2002)]. In the expressions below, the arguments in the functions $\xi (\theta ,\phi )$ and $h(\theta ,\phi )$ will not be explicitly written for the sake of brevity.

Variations of surface density can be related to the mass anomalies in terms of equivalent water heights (EWH) $\Delta H_w (\theta ,\phi )$ in terms of equivalent water heights (EWH) with a simple formula

10 $$\begin{aligned} \Delta H_\mathrm{w} (\theta ,\phi ) = \frac{\Delta \sigma (\theta ,\phi )}{\rho _\mathrm{w}}, \mathrm{where} \,\rho _w\, \mathrm{is\ water\ density}. \end{aligned}$$

Equations (5) and (8) describe the link between the temporal variations of surface density and the temporal variations of Stokes coefficients under the assumption that the Earth is a rigid body. In practice, solid Earth reacts elastically to changes in the load on the Earth’ surface (see, e.g., Boy and Chao 2005). Hence, actual variations in the gravitational potential comprise both the direct effect of mass transport and the elastic deformation of the Earth (deformation potential). In order to take this into account, additional scaling factors $(1+k_l)$ are introduced, where $k_l$ are load Love numbers (Wahr et al. 1998), so that Eq. (8) turns into

11 $$\begin{aligned} \Delta I_l (\theta ,\phi ) \approx (1+k_l) \left( 1 + \frac{\xi + h}{a} \right) ^{l+2} \Delta \sigma (\theta ,\phi ). \end{aligned}$$

The function $\Delta \sigma (\theta ,\phi )$, as any other function of coordinates $(\theta ,\phi )$, can be represented in terms of the spherical harmonic expansion. After retaining the spherical harmonic degrees consistently with Eq. (1), we have:

12 $$\begin{aligned} \Delta \sigma (\theta ,\phi ) = a \rho _\mathrm{w} \sum _{l^{\prime }=1}^{L_{\max }} \sum _{m^{\prime }=-l^{\prime }}^{l^{\prime }} \Delta \tilde{C}_{l^{\prime }m^{\prime }} \bar{Y}_{l^{\prime }m^{\prime }}(\theta ,\phi ), \end{aligned}$$

where $\Delta \tilde{C}_{l^{\prime }m^{\prime }}$ are Fourier coefficients, which can be computed on the basis of the Stokes coefficients $\Delta C_{lm}$. To that end, we insert Eqs. (12) and (11) into Eq. (5), which yields:

13 $$\begin{aligned} \Delta C_{lm}= & {} \frac{a^3\rho _\mathrm{w}}{M_{\text {E}}} \frac{(1+k_l)}{(2l+1)} \int \int \limits _{ \Omega } \bar{Y}_{lm} (\theta ,\phi ) \left( 1 + \frac{\xi + h}{a} \right) ^{l+2} \nonumber \\&\times \sum _{l^{\prime }=1}^{L_{\max }} \sum _{m^{\prime }=-l^{\prime }}^{l^{\prime }} \Delta \tilde{C}_{l^{\prime }m^{\prime }} \bar{Y}_{l^{\prime }m^{\prime }}(\theta ,\phi ) \, \hbox {d}\Omega . \end{aligned}$$

By interchanging the order of the summation and integration, we readily obtain:

14 $$\begin{aligned} \Delta C_{lm} = \frac{4\pi a^3\rho _\mathrm{w}}{M_{\text {E}}} \frac{(1+k_l)}{(2l+1)} \sum _{l^{\prime }=1}^{L_{\max }} \sum _{m^{\prime }=-l^{\prime }}^{l^{\prime }} B_{l,m,l^{\prime },m^{\prime }} \Delta \tilde{C}_{l^{\prime }m^{\prime }} \end{aligned}$$

with

15 $$\begin{aligned} B_{l,m,l^{\prime },m^{\prime }}= & {} \frac{1}{4\pi } \int \int \limits _{ \Omega } \bar{Y}_{lm} (\theta ,\phi ) \,\, \bar{Y}_{l^{\prime }m^{\prime }}(\theta ,\phi ) \nonumber \\&\quad \times \left( 1 + \frac{\xi + h}{a} \right) ^{l+2} \, \hbox {d}\Omega . \end{aligned}$$

Equation (14) represents a system of linear equations with constant coefficients $B_{l,m,l^{\prime },m^{\prime }}$, which form a square matrix. By solving this system, one can transform the Stokes coefficients into the coefficients $\Delta \tilde{C}_{l^{\prime }m^{\prime }}$. The system can be simplified further under some assumptions about the geometry of the Earth’s surface, as discussed below.

#### Spherical Earth approximation (radius $=$ 6378 km)

Let us assume that the Earth is the sphere of radius *a*, i.e., $r_s(\theta ,\phi ) = a$, $\xi (\theta ,\phi ) = 0$, and $h(\theta ,\phi ) = 0$. Then, in view of the fact that the surface spherical harmonics form an orthogonal set on a sphere, the matrix formed by coefficients $B_{l,m,l^{\prime },m^{\prime }}$ turns into the unit one:

16 $$\begin{aligned} B_{l,m,l^{\prime },m^{\prime }} = \delta _{l,l^{\prime }}\delta _{m,m^{\prime }}, \end{aligned}$$

where $\delta _{i,k}$ is the Kronecker delta. Furthermore, the Earth’s mass can be related to its mean density $\rho _{\text {E}}$:

17 $$\begin{aligned} M_{\text {E}} = \frac{4}{3} \pi a_{\text {E}}^3 \rho _{\text {E}}, \end{aligned}$$

where $a_{\text {E}}$ is the mean Earth’s radius. Then, one can insert Eqs. (17) and (16) into (14). If the difference between $a_{\text {E}}^3$ and $a^3$ is neglected (which is of the order of 0.3%), this readily results in:

18 $$\begin{aligned} \Delta C_{lm} = \frac{3\rho _\mathrm{w}}{\rho _{\text {E}}} \frac{(1+k_l)}{(2l+1)} \Delta \tilde{C}_{lm}. \end{aligned}$$

Thus, the computation of the coefficients $\Delta \tilde{C}_{lm}$ reduces to a scaling of the Stokes coefficients. A combination of Eqs. (18), (12), and (10) yields the well-known expression proposed for GRACE data processing by Wahr et al. (1998):

19 $$\begin{aligned} \Delta H_\mathrm{w} (\theta ,\phi ) = \frac{a}{3} \frac{\rho _{\text {E}}}{\rho _\mathrm{w}} \sum _{l=1}^{L_{\max }} \sum _{m=-l}^{l} \frac{(2l+1)}{(1+k_l)} \Delta C_{lm} \bar{Y}_{lm}(\theta ,\phi ). \end{aligned}$$

#### Ellipsoidal Earth approximation

Let the Earth’s surface geometry be an ellipsoid of rotation. Then, $h(\theta ,\phi ) = 0$ and $\xi (\theta ,\phi ) = \zeta (\theta )$, where $\zeta (\theta )$ is the height of the ellipsoid above the sphere of radius *a*. The radial distance $r(\theta )$ of the points at the ellipsoidal surface is given by:

20 $$\begin{aligned} r(\theta ) = a+\zeta (\theta ) = a \frac{1-f}{\sqrt{1-e^2\sin ^2\theta }}, \end{aligned}$$

where *f* is the ellipsoid flattening (WGS84 value: $f=1/298.2572$) and *e* is eccentricity $(e^2=2f-f^2)$. Then, Eq. (15) turns into

21 $$\begin{aligned} B_{l,m,l^{\prime },m^{\prime }}= & {} \frac{1}{4\pi } \int \int \limits _{ \Omega } \bar{Y}_{lm} (\theta ,\phi ) \bar{Y}_{l^{\prime }m^{\prime }}(\theta ,\phi ) \nonumber \\&\quad \times \left( \frac{1-f}{\sqrt{1-e^2\sin ^2\theta }} \right) ^{l+2} \, \hbox {d}\Omega . \end{aligned}$$

Furthermore, the trigonometric functions $\sin m \phi$ and $\cos m \phi$ form an orthogonal set in the interval $[0; 2\pi ]$:

22 $$\begin{aligned} \left\{ \begin{array}{l} \displaystyle \int _0^{2\pi } \sin m \phi \cdot \cos m^{\prime } \phi \, \hbox {d}\phi = 0 \\ \displaystyle \int _0^{2\pi } \cos m \phi \cdot \cos m^{\prime } \phi \, \hbox {d}\phi = \int _0^{2\pi } \sin m \phi \cdot \sin m^{\prime } \phi \,\, \hbox {d}\phi \\ \qquad = \pi (1+\delta _{m,0}) \cdot \delta _{m,m^{\prime }}. \end{array} \right. \end{aligned}$$

In view of Eq. (6), this allows the expression for the elements $B_{l,m,l^{\prime },m^{\prime }}$ to be simplified to:

23 $$\begin{aligned} B_{l,m,l^{\prime },m^{\prime }}= & {} \frac{(1+\delta _{m,0}) \cdot \delta _{m,m^{\prime }}}{4} \nonumber \\&\times \int _0^{\pi } \bar{P}_{l,|m|} (\cos \theta ) \cdot \bar{P}_{l^{\prime },|m^{\prime }|} (\cos \theta ) \sin \theta \nonumber \\&\times \left( \frac{1-f}{\sqrt{1-e^2\sin ^2\theta }} \right) ^{l+2} \hbox {d}\theta \nonumber \\= & {} \frac{(1+\delta _{m,0}) \cdot \delta _{m,m^{\prime }}}{4} \nonumber \\&\times \int _{-1}^{1} \bar{P}_{l,|m|} (x) \cdot \bar{P}_{l^{\prime },|m^{\prime }|} (x) \,\nonumber \\&\times \left( \frac{1-f}{\sqrt{1-e^2 (1-x^2)}} \right) ^{l+2} \, \mathrm{d}x. \end{aligned}$$

Thus, the system of linear equations given by Eq. (14) becomes block-diagonal and can be solved with ease.

#### Spherical Earth approximation (arbitrary radius)

Finally, one can also approximate the Earth’s surface with a sphere of an arbitrary radius: $r_s(\theta ,\phi ) = a + \Delta r$ (i.e., $\xi (\theta ,\phi ) = \Delta r$, $h(\theta ,\phi ) = 0$), with $\Delta r$ being an arbitrary value, provided that $\Delta r > -a$. In that case, the expression for the elements $B_{l,m,l^{\prime },m^{\prime }}$ simplifies to:

24 $$\begin{aligned} B_{l,m,l^{\prime },m^{\prime }} = \delta _{l,l^{\prime }}\delta _{m,m^{\prime }} \left( 1 + \frac{\Delta r}{a} \right) ^{l+2}, \end{aligned}$$

so that the link between the coefficients $\Delta \tilde{C}_{lm}$ and the Stokes coefficients is:

25 $$\begin{aligned} \Delta C_{lm} = \frac{4\pi a^3\rho _\mathrm{w}}{M_{\text {E}}} \frac{(1+k_l)}{(2l+1)} \left( 1 + \frac{\Delta r}{a} \right) ^{l+2} \Delta \tilde{C}_{lm}. \end{aligned}$$

The parameter $\Delta r$ can be chosen for a given target region such that the difference between the actual radial distance of the points at the Earth’s surface and the Earth’s equatorial radius *a* is taken into account.

One step further is a “locally spherical” approximation. With this, we mean that the parameter $\Delta r$ can be chosen not as a single constant for the entire target region, but as a value dependent on the location of the current point where the mass anomaly is computed. Let us assume, for example, that mass transport takes place at the ellipsoid, i.e., $\Delta r = \Delta r(\theta ) = \zeta (\theta )$. Then, a combination of Eqs. (25), (20), (12), and (10) yields the following expression for the mass anomalies in terms of EWH:

26 $$\begin{aligned} \Delta H_\mathrm{w} (\theta ,\phi )= & {} \frac{M_{\text {E}}}{4\pi a^2 \rho _\mathrm{w}} \sum _{l=1}^{L_{\max }} \sum _{m=-l}^{l} \frac{(2l+1)}{(1+k_l)} \, \Delta C_{lm} \nonumber \\&\quad \times \left( \frac{\sqrt{1-e^2\sin ^2\theta }}{1-f} \, \right) ^{l+2} \bar{Y}_{lm}(\theta ,\phi ) \end{aligned}$$

or, in view of Eq. (17),

27 $$\begin{aligned} \Delta H_\mathrm{w} (\theta ,\phi )= & {} \frac{a}{3} \left( \frac{a_{\text {E}}}{a} \right) ^3 \frac{\rho _{\text {E}}}{\rho _\mathrm{w}} \sum _{l=1}^{L_{\max }} \sum _{m=-l}^{l} \frac{(2l+1)}{(1+k_l)} \, \Delta C_{lm}\nonumber \\&\quad \times \left( \frac{\sqrt{1-e^2\sin ^2\theta }}{1-f} \, \right) ^{l+2} \bar{Y}_{lm}(\theta ,\phi ). \end{aligned}$$

This expression resembles Eq. (19), but contains an additional degree-depended scaling factor $\left( \frac{a_{\text {E}}}{a} \right) ^3 \cdot \left( \frac{\sqrt{1-e^2\sin ^2\theta }}{1-f} \, \right) ^{l+2}$.

The most appropriate choice of the Earth’s geometry approximation is discussed below.

### Selection of the most appropriate approximation of the Earth’s surface geometry

In the previous section, we presented a number of alternative expressions to convert temporal variations of Stokes coefficients into mass anomalies. The complexity of the associated computations depends on the adopted approximation of the Earth’s shape. In order to identify the most appropriate computational scheme, we consider the computation of mass anomalies from real GRACE data.

#### Data

In this study, we use monthly gravity field solutions in 2003–2015 produced at the Center for Space Research (University of Texas at Austin) (Bettadpur 2012). For a few months, the solutions are absent. Furthermore, we ignored the solutions that were not limited to a specific calendar month. As such, 135 monthly solutions in total are exploited. Each of these solutions is formed by a set of Stokes coefficients complete to degree 96. No filtering is applied.

It is expected that mass anomalies at relatively short spatial scales are particularly sensitive to the deviations of the assumed Earth’s surface geometry from the actual one. Therefore, it makes sense to focus on the scenarios where the high-frequency signal in GRACE data is strong, whereas the noise level is low. In view of this, we use the time series of Stokes coefficients to estimate the mean rate of linear mass change (i.e., linear trend) in the entire time interval 2003–2015. In this way, random noise in the coefficients is largely suppressed. The linear trend is co-estimated with a bias and a quadratic term, as well as with annual and semiannual (co-)sinusoidal variations [see, e.g., Eq. (15) in Siemes et al. (2013), for more detail].

#### Computation of mass anomalies at the actual Earth’s surface

As a reference, we convert the temporal variations of Stokes coefficients into mass anomalies using Eqs. (14) and (15) explicitly. To define the elevations $h(\theta ,\phi )$ of the Earth’s surface, we use the Global Land One-kilometer Base Elevation (GLOBE) digital elevation model (GLOBE Task Team et al. 1999). For inland areas, the model provides terrain elevations above the mean sea level. The oceans are flagged; we set the elevations there equal to zero. The geoid heights $\xi (\theta ,\phi )$ above the sphere of radius *a* are approximated by Eq. (20) for the ellipsoid (thus, we ignore the differences between the reference ellipsoid and geoid as relatively minor, $\lesssim ~100\hbox {m}$).

**Fig. 1:**
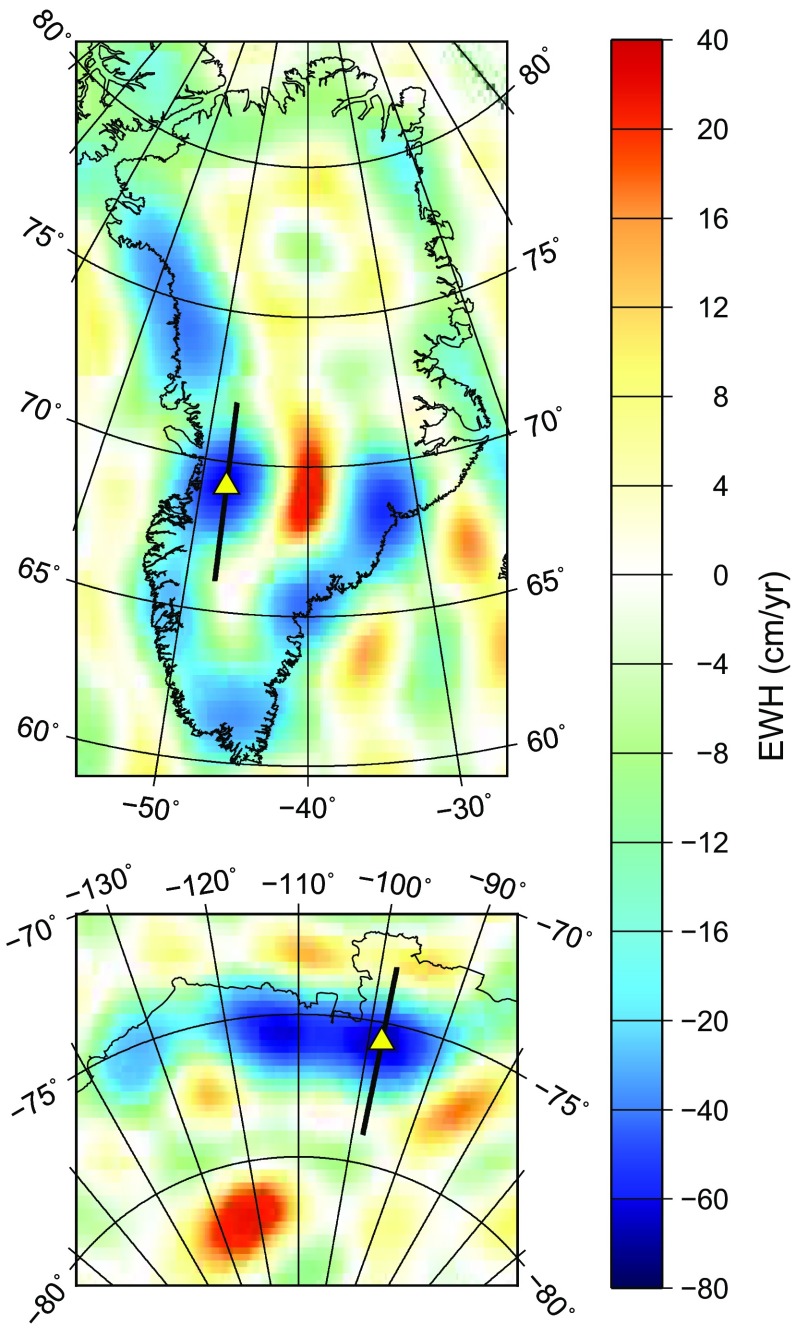
Mean rate of linear mass change in Greenland (top) and Amundsen Sea embayment of West Antarctica (bottom) in 2003–2015, in terms of EWH. The estimates are based on GRACE Release-5 monthly solutions produced at CSR. The mass anomalies are computed at the actual Earth’s surface, using GLOBE digital elevation model. Yellow triangles denote the locations of the peak signal: the Jakobshavn Isbræ in Greenland and the Pine Island glacier in West Antarctica. Thick black lines denote the profiles addressed in Fig. 5

In general, the computed mass change rates are heavily contaminated by random noise, which is not surprising in view of the absence of filtering (not shown). Nevertheless, noise in polar areas is relatively low and allows one to clearly see strong signals over the territory of Greenland and the Amundsen Sea embayment of West Antarctica (Fig. 1). The strongest negative trends are observed at the Jakobshavn Isbræ (West Greenland) and at the Pine Island glacier (West Antarctica), see Fig. 1 and Table 1. A rapid ice mass loss at both locations is also revealed by other observation techniques (e.g., Groh et al. 2014; Mouginot et al. 2014).

**Table 1:** Mass change rates (in terms of EWH) computed at the actual Earth’s surface, as well as the errors introduced by different assumptions about its geometry. The considered points are the locations of the peak signal

	Jakobshavn Isbræ (Greenland)	Pine Island glacier (West Antarctica)
Peak signal location		
Longitude	47$^{\circ }$W	98$^{\circ }$W
Latitude	69$^{\circ }$N	76$^{\circ }$S
Signal	− 64.0 cm/year	− 68.6 cm/year
Error of the spherical approximation ($R=6378$ km)	9.2 cm/year (14.4%)	9.7 cm/year (14.2%)
Error of the ellipsoidal approximation	− 1.0 cm/year (− 1.5%)	− 0.4 cm/year (− 0.6%)
Error of the (ellipsoid-based) locally spherical approximation	− 1.2 cm/year (− 1.8%)	− 0.6 cm/year (− 0.8%)
Difference between the locally spherical and ellipsoidal approximation	− 0.2 cm/year (− 0.3%)	− 0.2 cm/year (− 0.2%)

We explain the low noise level in Fig. 1, in spite of the absence of any filtering, by a combination of several factors. First, using 13 years of data allows random noise to be largely averaged out, as it is already mentioned above. Second, a large density of GRACE ground tracks, as well as the intersection of ascending and descending tracks at relatively large angles, ensures a good coverage of polar areas, which reduces random noise further. Third, Greenland and West Antarctica are notorious for the presence of very strong negative trends due to a rapid ice mass loss. Ironically, these locations are far away from the equator, so that deviations of the actual Earth’s surface from the sphere of radius $a\approx 6378$ km are large there. Thus, the presented areas can be considered as the “worst-case scenarios” for Eq. (19), which is traditionally used to convert Stokes coefficients into mass anomalies. Therefore, our further analysis is limited to the two geographical areas shown in Fig. 1.

#### Computation of mass anomalies at the sphere of 6378-km radius

**Fig. 2:**
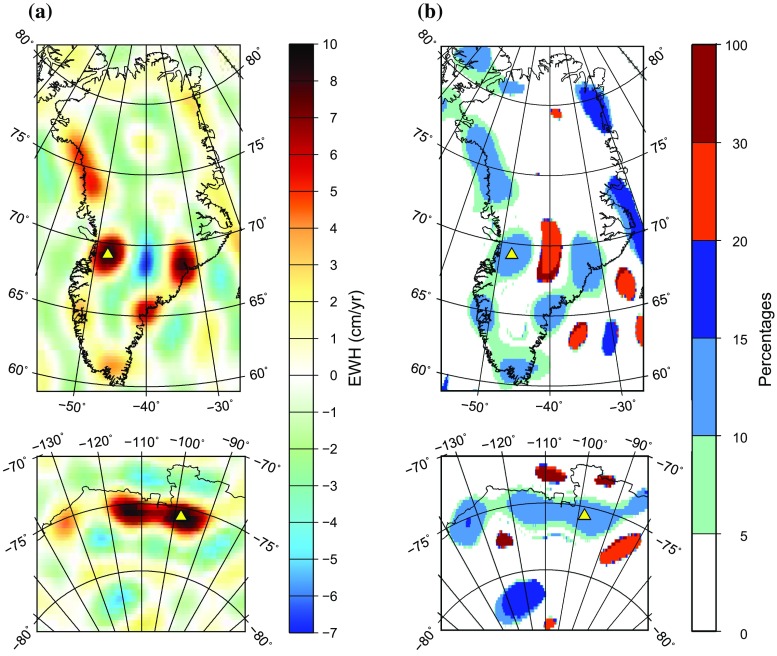
Difference (left) and the absolute value of relative difference in percentages (right) between the mass change rates computed at the sphere of radius $a=6{,}378{,}136$ m and those computed at the actual Earth’s surface. The relative differences are shown only at the locations where the signal exceeds in absolute value 10 cm/year. For more details, see the caption of Fig. 1 and the main text

**Fig. 3:**
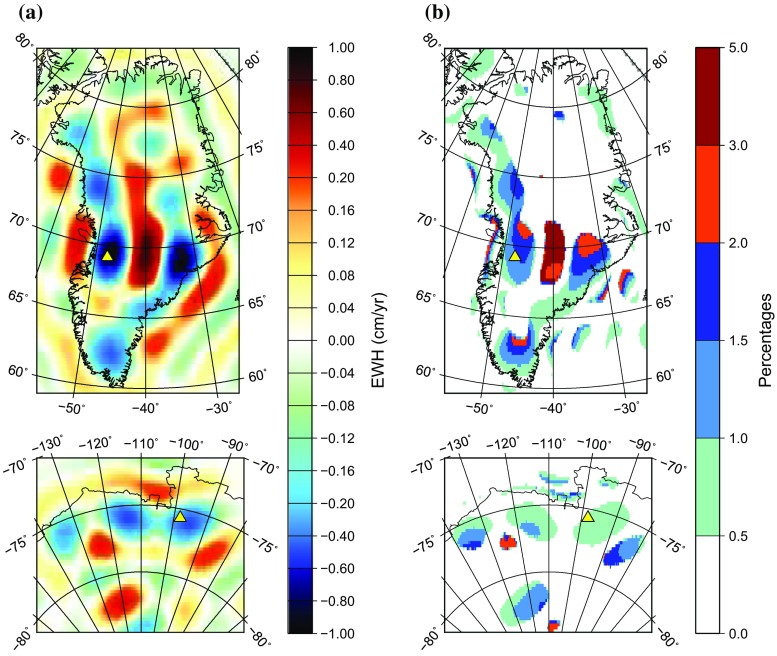
Same as Fig. 2, but the compared mass change rates are computed at the reference ellipsoid and at the actual Earth’s surface

**Fig. 4:**
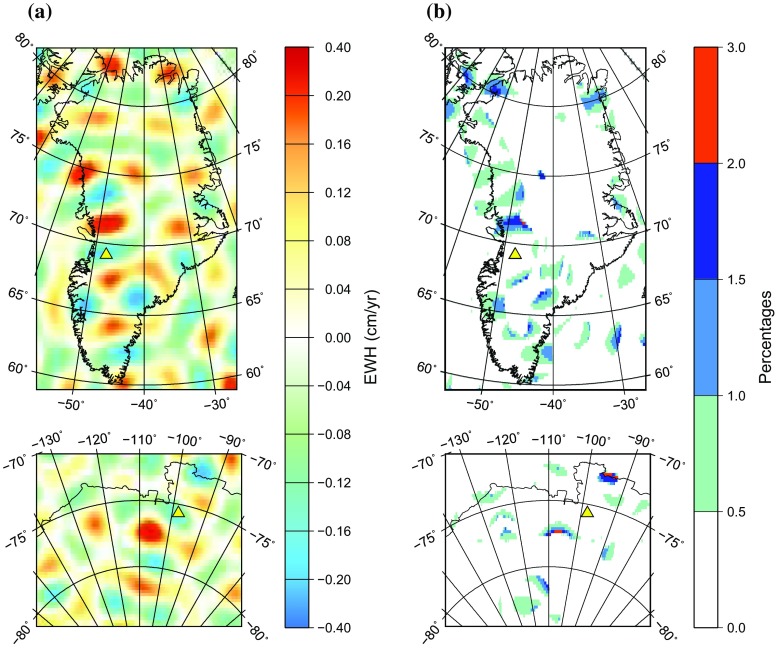
Same as Fig. 2, but the compared mass change rates are computed at the reference ellipsoid: using a locally spherical approximation and explicitly

Conversion of Stokes coefficients into mass anomalies with the commonly used Eq. (19) reveals large differences from the values computed at the actual Earth’s surface (see Fig. 2a). The observed differences are clearly anti-correlated with the total signal shown in Fig. 1. In other words, the traditionally used expression damps the recovered signals. This is not surprising, since the polar Earth’s radius is 21 km smaller than the equatorial one, so that actual mass transport in the considered case takes place about 20 km further away from GRACE satellites than it is implicitly assumed in the traditional computations. The largest differences reach about 10 cm/year (Fig. 2a, Table 1).

In order to better quantify the observed signal damping, we also represent the difference $D(\theta ,\phi )$ between the two variants of mass change rates in percentages, for which purpose the following expression is used:

28 $$\begin{aligned} D(\theta ,\phi ) = \frac{\left( \dot{H}_\mathrm{w}^{\mathrm{(appr)}}(\theta ,\phi ) - \dot{H}_\mathrm{w}^{\mathrm{(ref)}}(\theta ,\phi )\right) }{\left| \dot{H}_\mathrm{w}^{\mathrm{(ref)}}(\theta ,\phi )\right| } \times 100, \end{aligned}$$

where $\dot{H}_\mathrm{w}^{\mathrm{(appr)}}$ is the mass change rate computed with the approximate formula and $\dot{H}_\mathrm{w}^{\mathrm{(ref)}}$ is the reference mass change rate. The results are presented in Fig. 2b. We only show the observed differences if the reference signal at a given point exceeds in absolute value 10 cm/year. In this way, we mask out the large relative differences that are caused by a small value in the denominator in Eq. (28). In spite of that, the observed relative differences are, in general, quite large. At the locations of strongest negative trends, they are of the order of 15% (Table 1), whereas at some other locations they are even larger, reaching 20% and more (Fig. 2b). One may argue that the largest relative differences may be associated with noise (e.g., over the ocean and, perhaps, over the inner part of Greenland). Mass anomaly estimates there must be filtered anyway, so that the errors introduced by the spherical Earth approximation are not critical. Nevertheless, even if we limit the discussion only to the coastal areas, the signal damping caused by the considered approximation is at the level of up to 10–15%, which is hardly acceptable.

#### Computation of mass anomalies at the reference ellipsoid

Next, we compute mass anomalies under the assumption that mass transport takes place at the reference ellipsoid. To that end, we invert the block-diagonal matrix given by Eq. (23). The resulting mass change rates are an order of magnitude closer to those computed at the actual Earth’s surface, as compared to those produced under the assumption that the Earth is a sphere of 6378 km radius. The differences do not exceed 1 cm/year (Fig. 3a, Table 1). The relative differences are within 1.5% at the locations of the peak signal and typically stay within the 3% limit elsewhere (Table 1, Fig. 3b). Thus, the approximation of the Earth surface geometry with the reference ellipsoid may improve the conversion accuracy by an order of magnitude, as compared to the traditional approximation with the sphere of radius *a* = 6378 km.

In addition, it is worth noticing that the spatial pattern of the observed differences shows a clear positive correlation with the signal itself (cf. Figs. 1 and 3a). In other words, the recovered signal is sharper than the actual one. Obviously, this is due to the fact that the surface of the reference ellipsoid (which is close to the sea level) is a few km further away from the GRACE satellites than the actual surface of ice sheets. Finally, relatively large differences are observed in the ocean areas at the western and southeastern coasts of Greenland. We relate them to a strong gradient of mass anomalies at the Greenland coasts. In view of a limited spectrum of the function $\Delta H_\mathrm{w} (\theta ,\phi )$, ringing artefacts associated with the Gibbs phenomenon must occur in those areas. As soon as recovered signal becomes sharper, these artefacts become more pronounced.

#### Computation of mass anomalies at the reference ellipsoid using the locally spherical approximation

Finally, we compute mass anomalies at the reference ellipsoid using the locally spherical approximation. That is, the spherical Earth’s surface expression is used in the computations, but the radius of the sphere is latitude-dependent. At each latitude, it is set equal to the distance between the reference ellipsoid and the center of the Earth, cf. Eq. (26). The obtained linear trend estimates are surprisingly close to those produced with the explicit procedure addressed in the previous section. The differences between the results do not exceed 0.4 cm/year; see Fig. 4a. The relative differences stay at the level of at most 1–2% (Fig. 4b). Thus, the locally spherical approximation of the reference ellipsoid ensures an almost the same conversion accuracy as the usage of the reference ellipsoid explicitly. On the other hand, the conversion based on the locally spherical approximation is easier to implement in practice, since it does not require solving any systems of linear equations.

**Fig. 5:**
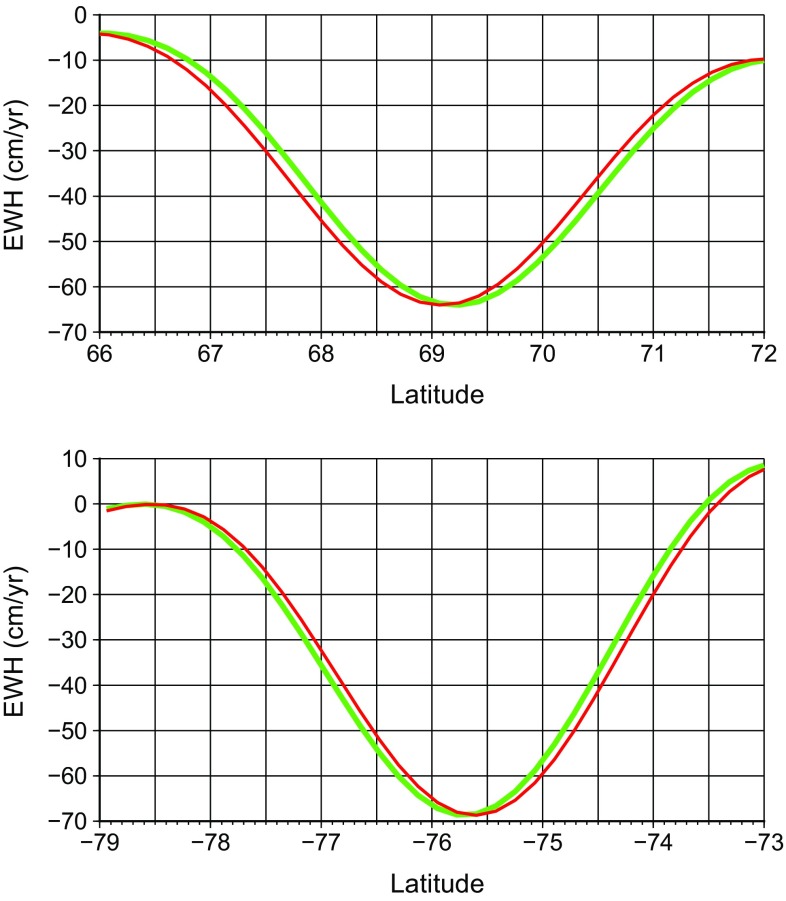
Mass change rates computed along the meridional profiles crossing the Jakobshavn Isbræ in Greenland (top) and the Pine Island glacier in West Antarctica (bottom); the exact location of the profiles is shown in Fig. 1. The computed values are presented as functions of geocentric latitude (in red) and geodetic latitude (in green). In both cases, it is assumed that mass transport takes place at the actual Earth’s surface, which is described by the GLOBE digital elevation model

## Ellipsoidal versus spherical coordinates

In this section, we discuss the difference between mass anomalies computed in spherical (geocentric) and ellipsoidal (geodetic) coordinates. The maximum difference between geodetic and geocentric (co-)latitudes is observed near the 45$^{\circ }$ latitudes, reaching approximately 11.5 arc-minutes or 21 km. In the polar areas, which are considered in our examples, this difference is smaller: of the order of 10–15 km or even less. One may argue that such differences must be negligible in the estimation of mass anomalies from GRACE data, since the spatial resolution of those estimates is a few hundreds of km. To demonstrate that such a statement may be unfair, we consider the meridional profiles that cross the Jakobshavn Isbræ in Greenland and the Pine Island glacier in West Antarctic (Fig. 5). The linear trend estimates along these profiles are presented as functions of both geodetic and geocentric latitude (Fig. 5). This figure clearly shows that the accuracy of locating a sharp signal may by far exceed the spatial resolution of GRACE data. As such, the difference between the estimates in spherical and ellipsoidal coordinates is clearly visible. Thus, we believe that the conversion of geodetic colatitudes into geocentric ones must not be omitted in GRACE data processing.

## Discussion and conclusions

To convert time-varying Stokes coefficients into mass anomalies at the Earth’s surface, geoscientists routinely use Eq. (19) or its equivalents. However, the results obtained with this expression represent mass transport at the spherical surface of 6378 km radius. In this study, we show that the accuracy of such a conversion may be insufficient, especially if the target area is located in a polar region and the signal-to-noise ratio is high. For instance, the mean linear trends in 2003–2015 estimated over Greenland and Amundsen Sea embayment of West Antarctica may be underestimated in this way by 10–15% or even more. Such an error may definitely exceed the noise level of current mass transport estimates. Moreover, there are no doubts that the accuracy and spatial resolution of future estimates will increase further, so that a limited accuracy of the conversion based on Eq. (19) will likely become in the future even less tolerable. There are several reasons to expect improvements in the mass anomaly estimation in the foreseeable future: (i) an ongoing progress in the techniques for satellite gravity data processing; (ii) a continuously increasing duration of mass anomaly time series, which leads to a high accuracy of mean estimates for the entire available time interval (including mean linear trends); and (iii) the forthcoming launch of the GRACE Follow-On (GFO) satellite mission (https://gracefo.jpl.nasa.gov). GFO satellites will be equipped with a laser interferometer, allowing for an order-of-magnitude increase in the accuracy of inter-satellite ranging. Though the increase in the ranging accuracy may not result in a proportional increase in the accuracy of estimated mass anomalies in general (Flechtner et al. 2016), the level-1 data will definitely become cleaner at high frequencies, since ranging noise at those frequencies is dominant (Flury et al. 2008; Ditmar et al. 2012). Then, this reduction in noise level will likely have a positive effect onto the estimates of mass anomalies at small spatial scales, which are particularly vulnerable if the Earth’s geometry is defined inaccurately.

As a solution, we propose an updated expression for the conversion of Stokes coefficients into mass anomalies. This expression is based on the assumptions that: (i) mass transport takes place at the Earth’s surface that is approximated by the reference ellipsoid; (ii) at each point of interest, the Earth’s surface is further approximated by the sphere with a radius equal to the current radial distance from the Earth’s center (“locally spherical approximation”). The updated expression is nearly as simple as Eq. (19), but allows the inaccuracies associated with the conversion procedure to be reduced by an order of magnitude.

In addition, we demonstrate that it is advisable to convert geodetic (co-)latitudes into geocentric ones, when mass anomalies are computed. This is in spite of the fact that the shifts caused by this conversion are an order of magnitude smaller than the spatial resolution of GRACE-based estimates.

In “Appendix A,” we summarize the recommended expressions for the conversion of Stokes coefficients into mass anomalies. Unlike in the main text, we use there the most traditional notation for the spherical harmonic expansion (when the orders start from 0 and not from $-l$), in order to facilitate the usage of the proposed expressions.

We would like to stress that the proposed conversion formula is particularly beneficial in the presence of strong signals in the range of high degrees, as it was the case in the considered examples. The absence of such signals makes the results much less sensitive to the assumption about the surface where mass transport takes place. For instance, the truncation of mass anomaly estimates at spherical harmonic degree 60 reduces the conversion errors 2–3 times, as compared to those presented in Fig. 2 (where the maximum degree is equal to 96).

In all the discussions so far, we assumed that the loading effects can be corrected just by introducing load Love numbers as additional scaling factors of the kind $(1+k_l)$. For a non-spherical Earth, such a simple approach is, strictly speaking, incorrect. However, the loading effects manifest themselves mostly in the range of low degrees. This can be understood from the fact that the scaling factors $(1+k_l)$ rapidly approach 1 as spherical harmonic degree increases. For instance, $(1+k_l)>0.96$ for degrees above 30 and $(1+k_l)>0.98$ for degrees above 70 (Wahr et al. 1998). Since the impact of the proposed conversion formula is mostly limited to the range of relatively high degrees, we believe that a simplified treatment of the loading effects is justified.

Finally, one may pose the questions whether mass anomalies can be uniquely restored considering that mass transport takes place at the Earth’s surface of a complicated geometry. For the case of a spherical Earth, a unique recovery of mass anomalies is guaranteed by Eq. (18). This formula establishes a unique link between the Stokes coefficients (that describe variations of gravitational potential) and the Fourier coefficients of mass anomalies. Moreover, this formula offers a practical way to make the corresponding conversion. By increasing the maximum spherical harmonic degree under consideration, one may, in principle, recover mass anomalies with an arbitrarily high spatial resolution. Thus, the spatial resolution of the results is fully defined by the spatial resolution of the exploited gravity field model. The situation with mass transport at the actual Earth’s surface is more complicated. First of all, the unique conversion of time-varying Stokes coefficients into mass anomalies can only be guaranteed if the matrix composed of coefficients $B_{l,m,l^{\prime },m^{\prime }}$ (cf. Eq. 15) is invertible. In all the computations conducted in this study, this was indeed the case: this matrix was not only invertible, but also close to the unit one. However, it remains unclear if (or under what conditions) this matrix remains invertible in general. Furthermore, the presence of nonzero off-diagonal elements in this matrix implies that there is no unique link anymore between the spatial resolution of gravity field model and that of mass anomalies. For instance, high-frequency signals in terms of mass anomalies can map onto low-frequency signals in gravity field observations. If the former signals contain spherical degrees above $L_{\max }$ in a given data processing run, a proper conversion of Stokes coefficients into mass anomalies becomes impossible. In other words, a realistic Earth’s geometry may result in a new type of high-frequency signal aliasing, which is absent when mass transport takes place at a spherical surface. A quantification of this effect and, if necessary, designing optimal schemes to mitigate it are the subjects of further studies.

## Appendix A: Recommended expressions to convert time-varying Stokes coefficients into mass anomalies at the Earth’s surface

1. Identify the time interval of interest. Subtract the mean value of each Stokes coefficient in this time interval from the coefficient time series to produce temporal variations of Stokes coefficients $(\Delta C_{lm}, \Delta S_{lm})$.
2. For each point of interest, convert the geodetic colatitude $\theta _g$ into the geocentric colatitude $\theta _c$:
  29 $$\begin{aligned} \theta _c = \text{ ATAN2 }\,(\sin \theta _g, \, (1-f)^2 \cos \theta _g), \end{aligned}$$
  where *f* is the flattening of the Earth’s figure (1 / 298.2572) and
  30 $$\begin{aligned} \text{ ATAN2 }(y,x) = \left\{ \begin{array}{ll} \arctan (y/x) &{}\quad \text{ if } x>0 \\ \arctan (y/x)+\pi &{}\quad \text{ if } x<0 \text{ and } y \ge 0 \\ \arctan (y/x)-\pi &{}\quad \text{ if } x<0 \text{ and } y< 0 \\ \pi /2 &{}\quad \text{ if } x=0 \text{ and } y > 0 \\ -\pi /2 &{}\quad \text{ if } x=0 \text{ and } y < 0 \\ \text{ undefined } &{}\quad \text{ if } x=0 \text{ and } y = 0. \\ \end{array}\right. \end{aligned}$$
3. For each point of interest, compute the mass anomaly $\Delta H_\mathrm{w}$ in terms of equivalent water heights using the following expression:
  31 $$\begin{aligned} \Delta H_\mathrm{w} (\theta _c,\phi )= & {} \frac{M_{\text {E}}}{4\pi a^2 \rho _\mathrm{w}} \sum _{l=1}^{L_{\max }} \frac{(2l+1)}{(1+k_l)} \,\nonumber \\&\times \left( \frac{\sqrt{1-e^2\sin ^2\theta _c}}{1-f} \, \right) ^{l+2} \end{aligned}$$
  32 $$\begin{aligned}&\times \left( \sum _{m=0}^{l} \Delta C_{lm} \bar{P}_{lm}(\cos \theta _c) \cos m \phi \, \right. \nonumber \\&\left. + \sum _{m=1}^{l} \Delta S_{lm} \bar{P}_{lm}(\cos \theta _c) \sin m \phi \right) , \end{aligned}$$
  where $\phi$ is the longitude of the current point; $M_{\text {E}}$ is the Earth’s mass ($5.9722 \times 10^{24}$ kg); *a* is the Earth’s equatorial radius (6, 378, 136 m); $\rho _\mathrm{w}$ is water density (1000 kg/m$^3$); $L_{\max }$ is the maximum spherical harmonic degree in the exploited GRACE data product; $k_l$ are load Love numbers (Wahr et al. 1998); $e^2=2f-f^2$, and $\bar{P}_{lm}(x)$ are normalized associated Legendre functions of argument *x*.
